# Macrophage Expression of Inflammatory Genes in Response to EMCV Infection

**DOI:** 10.3390/biom5031938

**Published:** 2015-08-18

**Authors:** Zachary R. Shaheen, John A. Corbett

**Affiliations:** Department of Biochemistry, Medical College of Wisconsin, 8701 W. Watertown Plank Rd, Milwaukee, WI 53226, USA; E-Mail: zshaheen@mcw.edu

**Keywords:** inflammation, cytokine, virus, macrophage, diabetes, Ccr5

## Abstract

The expression and production of type 1 interferon is the classic cellular response to virus infection. In addition to this antiviral response, virus infection also stimulates the production of proinflammatory mediators. In this review, the pathways controlling the induction of inflammatory genes and the roles that these inflammatory mediators contribute to host defense against viral pathogens will be discussed. Specific focus will be on the role of the chemokine receptor CCR5, as a signaling receptor controlling the activation of pathways leading to virus-induced inflammatory gene expression.

## 1. Introduction

Macrophages are a differentiated monocytic cell type within the innate immune system that have a well-defined role in the host response against viral infection [1,2]. Macrophages exert anti-viral effects through multiple mechanisms. They promote the removal and clearance of virally-infected cells, act as antigen presenting cells to shape the generation of an antigen-specific, adaptive immune response, and they express a range of effector molecules that promote anti-viral states and recruit other immune effectors to a local tissue environment. The macrophage responses to viral infection are important in the control and clearance of viruses, and have been described in detail by others [1,2]. In this review, the expression of inflammatory mediators which play a role in anti-viral immunity; specifically, the signaling mechanisms that regulate macrophage expression of inflammatory genes in response to viral infection will be addressed.

Macrophages express pattern recognition receptors (PRRs) that recognize foreign molecules (pathogen associated molecule patterns—PAMPs). The genetically inherited PRR families include Toll-like receptors (TLRs) and RIG-I-like receptors (RLRs) that recognize conserved structures that are specific to the type of pathogen (bacteria, virus, parasite) [3]. This recognition allows macrophages to produce a response that is selective for the PAMP.

For example, double stranded RNA (dsRNA), an intermediate formed during replication of many viruses, stimulates the production of anti-viral molecules including type I interferons (IFN) [3]. While significant progress has been made in identifying the PRRs that recognize PAMPS, and the pathways that control the selective innate immune responses to specific classes of invading pathogens, the importance of early and rapid induction of inflammation (chemokines, cytokines, lipid mediators and reactive oxygen and nitrogen species) in response to a specific class of pathogens, such as virus infection is not fully appreciated.

This review will focus on the activation of signaling cascades controlling the expression of the inflammatory genes interleukin (IL)-1, cyclooxygenase (COX)-2, and inducible nitric oxide synthase (iNOS) during virus infection in macrophages. The two isoforms of the pro-inflammatory cytokine IL-1 (α and β) are primarily secreted by activated macrophages and regulate local and systemic responses to injury and infection, including virus infection [4,5]. Local IL-1 production promotes changes in vascular endothelium to increase access of effector cells to sites of infection, and contributes to local tissue destruction, while systemic IL-1 promotes fever [4,5]. Additionally, IL-1 regulates T-cell polarization [4,5,6] and plays a crucial role in the production of antibodies in response to pathogens or adjuvants [6,7]. Prostaglandins, produced by COX-2 during infection, are soluble factors that are secreted by activated macrophages and participate in the regulation of viral infection [8]. Similar to IL-1, the prostagladin E_2_ (PGE_2_) induces fever, promotes edema, and facilitates immune cell infiltration to sites of infection [9,10]. iNOS has been defined as an anti-viral inflammatory gene, because of its ability to attenuate the replication of multiple viruses [11,12,13,14]. Viral clearance is reduced and the incidence in mortality is increased in mice lacking iNOS in response to viral infection [15]. Nitric oxide’s anti-viral effects may be explained, in part, by the S-nitrosation and inhibition of viral proteases [16], although this mechanism may not be generalizable to virus classes that do not require essential viral proteases to replicate. While IL-1, PGE_2_, and nitric oxide are all produced at high levels by macrophages following viral infection, these inflammatory mediators are not generally discussed as components of antiviral responses. The focus of this review will be on the mechanisms controlling the expression of IL-1β, COX-2, and iNOS, in macrophages during virus infection.

## 2. Encephalomyocarditis Virus (EMCV)

The non-enveloped, positive single-stranded RNA picornavirus EMCV has been used for many years to elucidate anti-viral activities of virus infected cells [17]. Further, infection of genetically susceptible mouse strains [17,18,19,20] with EMCV results in diabetes development by two mechanisms. At low doses (≤100 PFU), EMCV-induced diabetes requires macrophage activation and inflammatory mediator production [21]. It is the production of inflammatory cytokines (IL-1 and TNF) that contribute to the loss of insulin producing β-cells and diabetes development [21]. Macrophage depletion with silica [22,23] or anti- Mac1 and Mac2 antibodies [24,25] attenuates EMCV-induced diabetes, while inhibition or ablation of adaptive immunity does not modify the incidence of diabetes in response to EMCV infection. Specifically, thymectomized mice or mice treated with anti-lymphocyte serum are not protected from EMCV-induced diabetes, and it is not possible to transfer diabetes to recipient mice using lymphocytes or spleen cells isolated from EMCV-induced diabetic mice [26]. Furthermore, EMCV-infected mice lacking T-cells (athymic) or B-cells (µMT/µMT) become hyperglycemic in a manner similar to EMCV-infected controls [27]. Furthermore, depletion of helper T-cells with L_3_T_4_ antibodies and cytotoxic T-cells with Lyt2 antibodies does not modify diabetes incidence [24].

The mechanisms by which macrophages mediate the destruction of β-cells in EMCV-infected mice appear to be dependent on the production of inflammatory mediators. EMCV-induced diabetes in mice is attenuated by daily injection of neutralizing antibodies against the cytokines interleukin-1 (IL-1β) or tumor necrosis factor (TNF-α), or administration of aminoguanidine [28], a selective iNOS inhibitor [29,30]. *In vitro* studies using isolated rodent and human islets have shown that cytokines inhibit insulin secretion and kill β-cells by a mechanism that is associated with β-cell expression of iNOS and increased production of nitric oxide [31,32,33,34,35,36,37]. Because cytokines stimulate iNOS expression by β-cells [31,32,33], and EMCV-induced diabetes is attenuated by inhibitors of iNOS and antibodies that neutralize cytokines such as IL-1 and TNF [28], EMCV-induced diabetes is likely mediated by cytokine-stimulated iNOS expression and nitric oxide production by pancreatic β-cells that results in the loss of insulin secretion and β-cell viability. Consistent with this conclusion, EMCV-induced diabetes is attenuated in mice lacking iNOS [38]. These findings provide evidence to support a model in which inflammatory gene expression by activated macrophages contributes to the induction of diabetes following virus infection [39]. Inflammatory mediators, produced by macrophage, also participate in the development of diabetes in response to Kilham’s Rat Virus (KRV) infection in the Bio-Breeding (BB) rat [40,41,42,43], the natural development of diabetes in the NOD mouse [44,45], and other models of diabetes [46,47,48]. Because of the importance of macrophage soluble mediator production (cytokines, nitric oxide, prostaglandins) in the development of virus-induced diabetes, the pathways controlling the production of these soluble mediators by macrophages in the context of EMCV infection will be discussed.

## 3. Signaling Pathways Regulating Inflammatory Gene Expression in EMCV-Infected Macrophages

The transcription factor nuclear factor kappa B (NF-κB) plays a central role in the regulation of inflammatory genes [49]. NF-κB is normally held in the cytosol as an inactive dimer, bound to the inhibitory protein IκBα. Upon stimulation by pathogen associated molecular patterns (PAMPs), including Toll-Like Receptor (TLR) and double-stranded RNA (dsRNA) sensor ligands [3], IκBα is phosphorylated by Inhibitory Kappa Kinase (IKK). Phosphorylated IκBα is targeted for degradation in a proteasomal-dependent manner, releasing NF-κB to translocate to the nucleus and initiate transcription [49]. NF-κB plays a primary role in activating the expression of inflammatory genes in macrophages in response to treatment with synthetic double-stranded RNA (dsRNA-polyI:C) or EMCV infection. PolyI:C and EMCV stimulate IκB degradation, NF-κB nuclear localization, and NF-κB reporter activation in macrophages. Inhibitors of NF-κB attenuate NF-κB nuclear localization and the expression of inflammatory genes IL-1β, iNOS, and COX-2 in response to polyI:C and EMCV infection [50,51]. These findings are consistent with the known role of NF-κB in the regulation of iNOS [52,53], COX-2 [54,55], and IL-1β [56,57] transcriptional activation in response to other PAMPs, such bacterial endotoxin (lipopolysaccharide, LPS).

**Figure 1:**
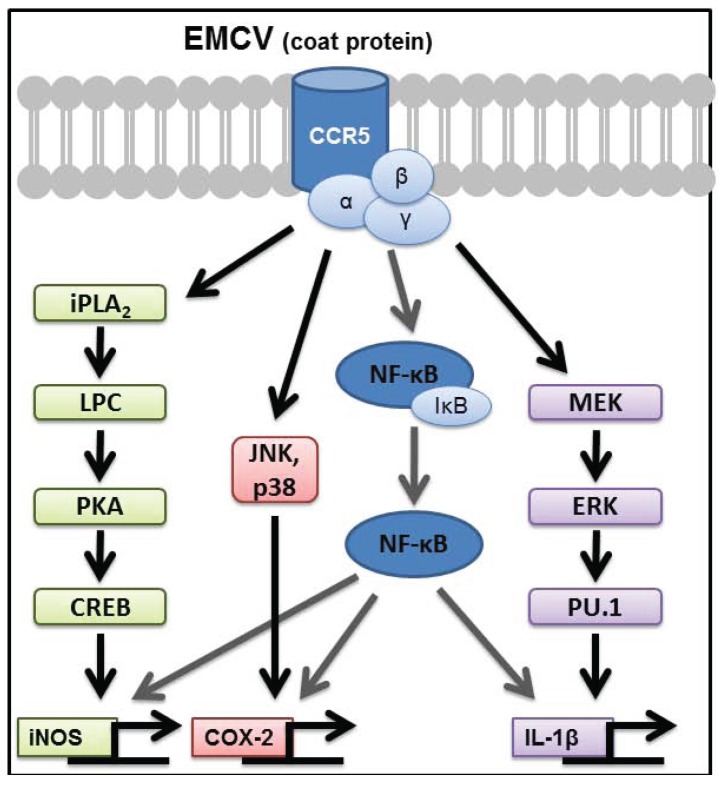
Signaling pathways required for encephalomyocarditis virus (EMCV) to stimulate pro-inflammatory gene expression in macrophages. The EMCV coat protein, devoid of viral RNA, activates CCR5-dependent signaling in macrophages within minutes of treatment. The transcription factor NF-κB is required for the expression of iNOS, COX-2, and IL-1β, and at least one additional pathway that is selective for one of these target genes. iNOS expression is controlled by iPLA2β and occurs through PKA-dependent activation of the transcription factor CREB. COX-2 expression is controlled by the MAP kinases p38 and JNK, whereas IL-1β expression is controlled by the MAP kinase ERK and transcription factor PU.1.

In addition to NF-κB, at least one secondary signaling pathway, selective for each target gene, is required for inflammatory gene expression by macrophages. These secondary signaling cascades include p38 and c-Jun N-terminal Kinase (JNK) for COX-2 expression [8,58], extracellular signal-regulated kinase (ERK) for IL-1 expression [59], and calcium independent phospholipase A2 (iPLA2β) and cAMP response element-binding protein (CREB) for iNOS expression [60,61,62] in response to EMCV infection or treatment with dsRNA (summarized in Figure 1). These secondary signaling pathways are selective for the specific target genes, as inhibition of p38 and JNK prevent polyI:C- and EMCV-stimulated COX-2 expression without modifying IL-1β or iNOS expression in macrophages [58]. Likewise, inhibition of ERK attenuates IL-1β expression without modifying iNOS or COX-2 [58,59]. ERK likely regulates IL-1β transcription via the transcription factor PU.1, similar to previously published work demonstrating LPS-induced IL-1β expression in an ERK and PU.1-dependent manner [63,64,65]. Inhibitors of iPLA2β and CREB attenuate iNOS expression without modifying IL-1 or COX-2 expression [60,61,62]. The regulation of CREB activation in response to EMCV infection and polyI:C treatment requires iPLA2β and PKA activation, yet occurs by a cAMP-independent pathway. We have identified a novel mechanism by which products of PLA_2_β activate PKA-dependent signaling independent of changes in the intracellular concentrations of cAMP [61,62]. Consistent with our studies, Williams and Ford showed that iPLA2β stimulates PKA-dependent CREB activation by a phospholipid-dependent, cyclic nucleotide-independent mechanism [66], including in polyI:C treated bovine pulmonary artery endothelial cells [60]. Lysophospholipids are one product of PLA_2_β that may mediate PKA activation based on the ability of this lipid to reconstitute iNOS expression in PLA_2_β inhibited macrophages treated with polyI:C [61]. Overall, these findings emphasize the selectivity in pathways required for the activation of inflammatory genes in response to virus infection of macrophages (summarized in Figure 1).

## 4. Additional Signaling Pathways That Contribute to the Regulation of Inflammatory Genes in EMCV-Infected Macrophages

While polyI:C, EMCV, or LPS alone is sufficient to stimulate the expression of iNOS in RAW264.7 macrophages, a second signal, IFN-γ is required to stimulate iNOS expression in naïve mouse macrophages [50,62]. IFN-γ stimulates the expression of interferon regulatory factor-1 (IRF-1), a transcription factor required for the expression of iNOS [67]. While IFN-γ is not required for polyI:C or EMCV-mediated COX-2 expression and production of prostaglandin PGE_2_, in the presence of IFN-γ, polyI:C and EMCV-stimulated COX-2 expression is enhanced in macrophages [51]. IL-1β expression in mouse macrophages occurs in response to polyI:C alone and is not enhanced by IFN-γ, although IFN-γ appears to promote the release of biologically active IL-1β [50] in an IRF-1-independent manner [67]. In RAW264.7 macrophage cell lines, polyI:C and EMCV are sufficient to stimulate the expression of iNOS, COX-2, and IL-1β and IFN-ү enhances this response [50,51,67]. 

In searching for mechanisms that would allow for the activation of each of the signaling cascades that selectively control the expression of inflammatory genes (iNOS, COX-2, and IL-1β) in macrophages, we showed that pharmacological inhibitors and dominant negative mutants of the Src family kinases (SFKs) attenuate EMCV-stimulated expression of iNOS, COX-2, and IL-1β in macrophages [68]. SFKs are non-receptor tyrosine kinases that have been implicated in the expression of iNOS, cytokines IL-1β and TNF-α, and COX-2 in response to LPS [69] and bacterial DNA (CpG) [70]. Inhibition of SFKs attenuates EMCV-stimulated IκBα degradation and phosphorylation of p38, but did not affect virus stimulated JNK or ERK phosphorylation [68]. These data suggest that SFKs regulate the expression of inflammatory genes, at least in part, through the activation of NF-κB. Consistent with a role of SFKs, Yoon and coworkers have shown that the SFK member Hck participates in the regulation of macrophage inflammatory genes in mice infected with EMCV [71].

One potential downstream target of SFKs is phosphoinositide-3 kinase (PI3K). PI3K is composed of an 85 kDa regulatory subunit (p85) and a 100 kDa catalytic subunit that when activated phosphorylates interior plasma membrane lipids [72]. These triphosphorylated inositide lipids serve as docking sites to promote the activation of signaling molecules that contain plekstrin homology (PH) domains. SFKs may promote PI3K activation through the PI3K regulatory subunit SH2 domain, which recognizes and binds to tyrosine-phosphorylation sites on SFKs, or through the two proline-rich regions on PI3K’s regulatory subunit that could interact with the SH3 domains on SFK [73,74]. While the direct effects of EMCV infection on SFK-mediated PI3K activation has not been examined, PI3K inhibition (pharmacologically or by transfection with dominant negative PI3K) attenuates EMCV- and polyI:C-stimulated expression of iNOS, COX-2, and IL-1β in a manner similar to conditions of SFK inhibition [75]. The inhibition of PI3K in EMCV-infected macrophages also changes their morphology from an activated inflammatory phenotype to apoptosis and this shift in morphology is associated with elevated levels of caspase-3 activity [75]. Consistent with our observations, PI3K inhibitors cause macrophage apoptosis in response to parainfluenza virus infection [76]. It is interesting to speculate that PI3K may determine the phenotypic response of macrophages to virus infection. When PI3K is activated in response to virus infection, macrophage activation results in the production of inflammatory mediators to combat the infection; however, if PI3K is not activated, virus infection can result in macrophage apoptosis. In addition to macrophage activation, PI3K-dependent signaling participates in the establishment of latent and chronic viral infections in host cells [77,78]. The differences in responses are likely to be cell type and virus specific.

## 5. Role of dsRNA Sensors in Response to Virus Infection

Picornaviruses, including EMCV, are positive, single-stranded RNA viruses that form double-stranded RNA (dsRNA) when replicating their genome [17]. dsRNA sensors are a diverse group of Pattern Recognition Receptors (PRRs) that control cellular responses to virus infection. The principle response directed by dsRNA sensors is the induction of type I interferons (IFNs) [79]. In addition to type I IFNs, dsRNA sensors have been reported to regulate the activation of pro-inflammatory signaling pathways (including NF-κB) and the expression of cytokines [3].

There are a number of dsRNA sensors, each with unique subcellular locations and functions that are activated in response to EMCV infection and synthetic dsRNA (polyI:C) treatment. These receptors include toll-like receptor TLR-3, melanoma differentiation associated protein-5 (mda-5), retinoic acid-inducible gene (RIG-I), and protein kinase R (PKR), one of the first dsRNA sensors to be identified. PKR is a cytoplasmic eIF2α kinase that is auto-phosphorylated following dsRNA binding and phosphorylates targets such as eIF2α leading to the inhibition of translation initiation [80,81,82]. In addition to the phosphorylation of eIF2α, PKR participates in the regulation of additional signaling cascades in a cell type selective manner. Included in these cascades is the MAP kinases pathways and NF-κB [81]. TLR-3 is a dsRNA sensor that is localized to endosomes [83] and the retinoic acid-inducible gene like receptors (RLRs) mda-5 and RIG-I are localized to the cytoplasm. Mda-5 is the primary cytosolic dsRNA sensor that responds to long dsRNA, including polyI:C and EMCV viral genome, while RIG-I is not activated in response to EMCV or polyI:C and instead selectively responds to short dsRNA or uncapped ssRNA [84,85,86]. The primary role of each of these dsRNA sensors is to increase the production of type I IFNs and the induction of a classic type I IFN response to a virus infection or polyI:C treatment. While dsRNA sensors function in the induction of type I IFN in infected cells, they are dispensable for the activation of inflammatory gene expression by macrophages. Macrophages isolated from wild-type mice and mice deficient in mda-5 [87], TLR-3 [87], or PKR [51,59,61,88] express iNOS, COX-2, and IL-1β to equal levels in response to EMCV infection or polyI:C. Furthermore, the signaling cascades required for the expression of each of the inflammatory genes are activated to similar levels in macrophages isolated from wild-type mice or mice deficient in these dsRNA sensors [87,88]. Much like macrophages, PKR does not appear to participate in dsRNA- or EMCV-induced IκBα degradation, NF-κB nuclear localization, and NF-κB promoter activity in PKR^−/−^-deficient fibroblasts [89].

In contrast to the induction of inflammatory gene expression, dsRNA sensors are required for the induction of a type I IFN response to EMCV infection or polyI:C treatment. Macrophages lacking TLR-3 fail to express type I IFNs in response to polyI:C treatment and macrophages deficient in mda-5 fail to produce type I IFNs in response to EMCV infection [87]. Furthermore, in macrophages isolated from mice deficient in the type I IFN receptor (IFNα/βR), expression of iNOS and IL-1β in response to EMCV infection is comparable to macrophages isolated from wild-type mice [88]. The type I IFN response is important for controlling virus infection, as titers of virus that accumulated in mice lacking dsRNA receptors are elevated and mortality increased following EMCV infection [90,91]. While these findings are consistent with the well-studied role of dsRNA sensors as a classic antiviral response, the importance of the dsRNA-independent induction of inflammatory genes in response to EMCV infection and polyI:C treatment as an antiviral response is unclear.

The induction of inflammatory gene expression in response to EMCV infection appears to be independent from the induction of the classical antiviral type I IFN response. Overall, the two responses do not temporally overlap, as EMCV rapidly activates the signaling cascade required for iNOS, COX-2 and IL-1β expression within minutes of infection, while EMCV RNA accumulation is a slow process first detectable 4–6 hours post infection in macrophages [88]. Furthermore, the accumulation of RNA is not required for EMCV-stimulated inflammatory gene expression by macrophages. We have shown that EMCV capsid protein devoid of viral RNA is sufficient to stimulate the activation of pro-inflammatory signaling cascades and the expression of iNOS, COX-2, and IL-1β expression to levels similar to unaltered EMCV [87,88]. Because EMCV capsid protein is sufficient to activate signaling within minutes of treatment it is likely that pro-inflammatory signaling occurs through the activation of a cell-surface receptor.

## 6. CCR5 as the Signaling Receptor Responsible for Inflammatory Gene Expression in Response to EMCV Infection

The chemokine receptor CCR5 is a cell-surface G protein-coupled receptor (GPCR) that is expressed in macrophages, lymphocytes, and dendritic cells [92]. Endogenous ligands for CCR5 that promote chemotaxis include the chemokine ligands Ccl3, Ccl4, and Ccl5 (RANTES) [92,93,94]. CCR5 also functions as a co-receptor for macrophage-tropic HIV infection [95,96] by interacting with gp120 on the HIV envelope [97], and decreased CCR5 expression affords resistance to HIV infection [98,99]. Tyner and Holtzman have identified a role for CCR5 in promoting macrophage survival that is mediated by PI3K and MAPK activation during parainfluenza and influenza infection in mice [76]. We have shown that neutralization antibodies to CCR5 attenuate IκBα degradation, MAP kinase phosphorylation, and the expression of iNOS, COX-2, and IL-1β in EMCV-infected macrophages [87]. Consistent with CCR5 antibody neutralization, macrophages isolated from CCR5^−/−^ mice fail to express iNOS, COX-2, and IL-1β in response to EMCV infection (summarized in Figure 1). EMCV capsid protein, void of virus RNA, is sufficient to stimulate CCR5-dependent signaling and expression of inflammatory genes in macrophages [87]. Whereas CCR5 is required for the expression of inflammatory genes in response to EMCV infection of macrophages, CCR5 is not required for viral entry or replication, as EMCV RNA accumulates in infected macrophages harvested from CCR5^−/−^ mice [87]. These observations are consistent with previous work demonstrating that adhesion molecules (likely VCAM-1)—and not CCR5 —are the likely viral-entry receptors for EMCV and other picornaviruses [100,101,102,103]. The induction of inflammatory genes in response to EMCV infection appears to function as an antiviral response, as virus RNA accumulates to levels eight-fold higher in macrophages isolated from CCR5^−/−^ mice compared to macrophages isolated from wild-type control mice infected with EMCV [87]. The enhanced accumulation of EMCV RNA in macrophages isolated from CCR5 deficient mice is also associated with increased expression of type I IFNs [87].

CCR5 is a promiscuous receptor that either binds directly to, or is activated by, multiple ligands. In addition to its role in regulating chemokine signaling in response to Ccl3, Ccl4, and Ccl5 (RANTES) [92,93,94], CCR5-dependent signaling is also activated by bacterial heat shock protein Hsp70 [104]. CCR5 controls a number of the effector functions of dendritic cells including the expression of IL-6 in response to virus infection [104] and functions as a co-receptor that facilitates HIV entry during infection [95,96] by interacting with the HIV envelope protein gp120 [97]. Macrophages also express iNOS and COX-2 in response to HIV infection [105,106], and gp120 is required for HIV-stimulated-1β expression by macrophages by a pathway that is dependent on CCR5, SFKs, and PI3K [107,108]. CCR5 is also a signaling receptor that maintains macrophage viability responses during influenza and parainfluenza infection [76]. These findings are consistent with a role for CCR5 as an integral cell surface receptor that participates in the activation of inflammatory cascades in macrophage in response to virus infection and suggest that CCR5 may function as a Pattern Recognition Receptor that participates in the rapid activation of an inflammatory response.

We hypothesize that CCR5 activation may function in an early and rapid response to infection, where virus coat protein or products of bacteria (such as hsp 70) trigger the induction of inflammatory genes such as iNOS, COX-2 and IL-1β. The function of these gene products may be to inform neighboring cells that a pathogen is present (IL-1; [4,5]) resulting in the activation of pathways the limit infectivity or replication of the pathogen. This is known for nitric oxide [11,12,13,14,15,16] and prostaglandins [8,9,10,109] which inhibit virus replication. This affords cells in the vicinity of infection time to identify the pathogen and generate a more selective response based on the presence of a PAMP. In the case of virus infection, a dsRNA PRR would be activated and stimulate a type I IFN response [3,79,110,111]. This activation of dsRNA sensors that control the induction of type I IFNs does not require CCR5-dependent signaling [87,88]. In support of this hypothesis, we have shown that CCR5 does not participate in the induction of type I IFNs by virally infected macrophages, and that EMCV replication and the subsequent induction of type I IFNs is enhanced in mice lacking CCR5 [87].

## 7. Conclusions

**Figure 2:**
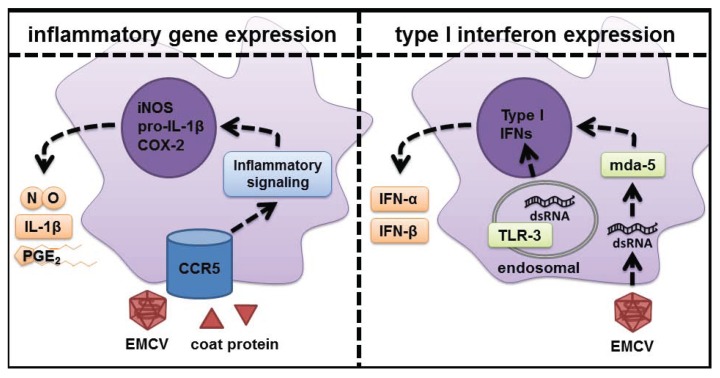
Differential responses of macrophages to EMCV infection. EMCV stimulates the activation of pro-inflammatory signaling within minutes and these pathways regulate the transcriptional activation of inflammatory genes (iNOS, COX-2, and IL-1β) during viral infection. This pro-inflammatory response does not require the presence of dsRNA sensors or dsRNA sensor-dependent type I IFN production. It is hypothesized that this inflammatory cascade serves to maintain macrophage survival, limit replication of the pathogen, and to inform neighboring cells that a pathogen is in the vicinity. These events afford time for the innate immune response to identify the pathogen through a PRR and activate a more selective, secondary response that in the case of viral infection includes the expression and production of type I IFNs in response to viral dsRNA replicative intermediates.

The studies outlined in this review provide evidence that CCR5 functions as an integral cell surface signaling receptor that participates in signaling events leading to the expression of the inflammatory genes iNOS, COX-2, and IL-1β by macrophages in response to EMCV infection. This CCR5-dependent response is activated within minutes of EMCV infection, and both temporally precedes and is mechanistically dissociated from the type I interferon response, which is not induced until hours later when EMCV RNA replication is detectable within macrophages (Figure 2). The activation of CCR5 by EMCV coat protein is consistent with the activation of this receptor by additional PAMPs from multiple virus classes and bacteria, which have been shown to stimulate CCR5-dependent responses. While this response does not appear to be a specific PRR/PAMP activated signaling cascade, activation of CCR5 signaling by EMCV coat protein appears to function as an anti-viral response. This cascade leads to an attenuation in the replication of EMCV allowing additional time for the innate immune systemt to generate a more specific PRR-dependent response that is selective to the type of infection, which in the case of virus infection results in the induction of type I interferons.
